# A novel clinical method to measure skin staining reveals activation of skin damage pathways by cigarette smoke

**DOI:** 10.1111/srt.13108

**Published:** 2021-11-10

**Authors:** Annette Dalrymple, Michael McEwan, Marianne Brandt, Stephan Bielfeldt, Emma‐Jayne Bean, Alain Moga, Steven Coburn, George Hardie

**Affiliations:** ^1^ British American Tobacco, R&D Southampton Hampshire UK; ^2^ proDERM Institut für Angewandte Dermatologische Forschung Hamburg Germany; ^3^ Synelvia SAS Labége France

**Keywords:** cigarette smoke, cosmetic, electronic cigarette/e‐cigarette, hygiene, skin damage, skin staining, tobacco heating product

## Abstract

**Background:**

Long‐term use of cigarettes can result in localised staining and aging of smokers’ skin. The use of tobacco heating products (THPs) and electronic cigarettes (ECs) has grown on a global scale; however, the long‐term effect of these products’ aerosols on consumers’ skin is unknown. This pilot clinical study aimed to determine whether THP or EC aerosol exposure results in skin staining or activation of biomarkers associated with oxidative stress.

**Materials and methods:**

Eight areas were identified on the backs of 10 subjects. Two areas were used for air control, and two areas exposed to 32‐puffs of cigarette smoke (CS), THP or EC aerosols, which were delivered to the skin using a 3‐cm diameter exposure chamber and smoke engine. Skin colour was measured using a Chromameter. Squalene (SQ), SQ monohydroperoxide (SQOOH) and malondialdehyde (MDA) levels were measured in sebum samples by mass spectrometry and catalase colorimetry.

**Results:**

CS exposure significantly increased skin staining, SQOOH and MDA levels and SQOOH/SQ ratio. THP and EC values were significantly lower than CS; EC values being comparable to air control. THP values were comparable to EC and air control at all endpoints, apart from skin staining. SQ and catalase levels did not change with exposure.

**Conclusions:**

CS stained skin and activated pathways known to be associated with skin damage. THPs and ECs produced significantly lower values, suggesting they could offer hygiene and cosmetic benefits for consumers who switch exclusively from smoking cigarettes. Further studies are required to assess longer‐term effects of ECs and THPs on skin function.

**Abbreviations**
ANOVA: a one‐way analysis of variance
CIE: (Commission Internationale de L'éclairage)
CORESTA: Cooperation Centre for Scientific Research Relative to Tobacco
CRM No 81: CORESTA recommended method No 81
CS: cigarette smoke
EC: electronic cigarette
HCI: health Canada intense
SD: standard deviation
THP: tobacco heating product
Δ: delta

## 1 INTRODUCTION

Electronic cigarettes (ECs) and more recently tobacco heating products (THPs) have increased in popularity; however, their availability varies globally due to local regulations. ECs function to heat an e‐liquid, composed from propylene glycol, vegetable glycerol, water, flavours and nicotine, into an inhalable aerosol. THPs are used with tobacco consumables/sticks, and the device heats the tobacco within the consumable to 200−350°C. Heating vaporises nicotine and other volatile compounds within the tobacco consumable, but the temperature limit ensures the tobacco does not burn, as occurs in a lit cigarette that reaches temperatures of 950°C. A lit cigarette also smoulders between puffs, producing side‐stream smoke; THPs and ECs only produce an aerosol when the consumer puffs on the heated tobacco consumable or EC device.

The lower temperature profiles of THPs, and the absence of tobacco from the majority of e‐liquids, result in aerosols with significantly lower levels of toxicants compared to cigarette smoke (CS).^1^, ^2^, ^3^ Furthermore, when cells are exposed in vitro to THP or EC aerosols, reduced or no biological responses occur.^4^, ^5^, ^6^, ^7^, ^8^, ^9^ Clinical studies in which subjects switch to THPs or ECs or quit all forms of tobacco have also demonstrated that biomarker levels in switching subjects are comparable to quitters or non‐smokers.^10^, ^11^, ^12^, ^13^, ^14^, ^15^, ^16^

Many studies have linked CS to the staining of fingernails and facial hair, and smoking is also thought to cause greying of facial skin.^17^ Furthermore, CS can cause localised oxidative stress, and continued exposure can affect skin barrier integrity and connective tissue degeneration, leading to wrinkle formation.^17^, ^18^, ^19^, ^20^, ^21^ A study has suggested that smoking can age the skin by 30 years, and that facial wrinkles observed in a 40‐year‐old smoker resemble that of a 70‐year‐old non‐smoker.^22^ However, the effects of THP and EC use on a consumer's skin and the benefits of smokers switching to THPs and ECs are currently unknown.

In this pilot clinical study, 10 healthy non‐smokers without underlying skin or systemic disease were recruited. Eight areas on the scapular area of each subject's back were identified and exposed to 32 puffs of CS, THP or EC aerosols; two untreated areas served as controls. Following exposure, colour was quantified, and the levels of Squalene (SQ), SQ monohydroperoxide (SQOOH), malondialdehyde (MDA) and catalase were measured in skin sebum samples.

## 2 MATERIALS AND METHODS

### 2.1 Products

All products used in this study were manufactured by British American Tobacco and are commercially available in a number of European Countries (Table 1). To enable blind testing, the brand was not printed on the cigarettes; however the number N491 was printed for product identification. The glo device and Classic Tobacco Neostik have previously been described in detail.^2^ ePen 3, a closed modular rechargeable EC, was used with a blended tobacco e‐liquid cartridge (18 mg/ml nicotine) and has previously been described in detail.^23^ All products and devices were stored at room temperature; glo and ePen 3 devices were charged daily before use.

**TABLE 1:** Products assessed

Product category	Product	BAT product code	Source	Puffs per product/cartridge	Puffing regime	Puff profile
Cigarette	Commercial cigarette blend	N491	BAT, UK	7	HCI^a^	Bell
Tobacco heating product	Glo and classic tobacco Neostik	THP1.0_LN1_05N0_K003	BAT, UK	8	HCI^m^	Bell
E‐cigarette	Vype ePen 3 and blended tobacco e‐liquid 18 mg/ml nicotine	PEN3.0BT18	BAT, UK	32	CRM81	Square

Abbreviation: CRM81, Cooperation Centre for Scientific Research Relative to Tobacco recommended method no 81 (2015); HCI, health Canada intense smoking regime (Health Canada, 1999); HCIm, health Canada intense smoking regime modified with no vent blocking.

^a^ Vents blocked 100% on product.

### 2.2 Study design

**FIGURE 1:**
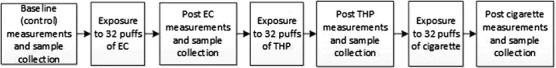
Overview of product exposure and sample collection

This study was reviewed and approved by the Institutional Review Board of proDERM GmbH. Written informed consent was obtained from all individual subjects prior to their participation in the study and before undergoing any study procedures, including screening assessments. The study was conducted approximating the main principles of the ICH Guideline for Good Clinical Practices at a single centre in Schenefeld, Hamburg. An overview of the clinical assessment is detailed in Figure 1.

### 2.3 Selection of study participants

For screening, 10 subjects came to the study site, were informed about the study and gave written consent. Subjects’ medical history, concomitant therapies and eligibility according to the study inclusion/exclusion criteria (Table S1) were recorded by the study physician. Three months prior to the study date, all subjects were asked to refrain from sun exposure, UV‐therapy and/or artificial tanning in the test area. Three days prior to the assessment day, all subjects were required to refrain from applying any leave‐on cosmetics (e.g., creams, lotions, oily cleansing products) to the test area, and not apply any detergents (e.g., soaps, shampoos, bath and shower products) to the test area on assessment day.

### 2.4 Assignment of test areas

Eight test areas were assigned (two per product or untreated control) on the back of each subject. Areas were assigned in two rows of four test areas, starting on the left upper side of the back (area 1) and finishing towards the lower right side of the back (area 8). Test areas were assigned on the upper part of the back in the lipid‐rich T‐zone. The fields near the spine were used for lipid sample collection (areas 2, 3, 6 and 7), and the fields away from the spine were used for colour measurements (areas 1, 4, 5 and 8). Treatments were assigned to the test areas according to a 4 × 4 orthogonal Latin Square with randomly permuted blocks of fixed size.

### 2.5 Product exposure

Product exposure and instrumental measurements took place in an air‐conditioned room at 21 ± 1°C and 50% ± 5% relative humidity. Before measurements, subjects acclimatized in the room for a minimum of 30 min. Control colour measurements and control lipid samples were collected as detailed below before any product exposure.

A 3‐cm diameter exposure chamber was fixed to the subject's back using self‐adhesive electrode pads (proDERM propriety information), as described previously.^23,24^ The exposure chamber was then attached to a Borgwaldt A14 smoke machine (Borgwaldt‐KC, Hamburg, Germany) and 32 puffs of each product delivered to the chamber. Specific puffing regimes were used (Table 1), and subject's skin exposed to the products in the following order: ePen 3, glo and N491 cigarette (Figure 1). Two skin areas per product were exposed to enable one area be assessed for colour and the other to have lipid biomarker levels assessed. Between each product, the exposure chamber was changed, the smoke machine cleaned, and plastic tubing from the smoke engine to both the chamber and exhaust changed.

### 2.6 Colour measurements

Prior to product exposure (untreated control) and after product exposure, the colour profile of one the designated skin areas was assessed using a Chromameter CR 400 (Minolta, Device D‐Langenhagen, Germany). The Chromameter measurement area was 8 mm in diameter, and four independent measurements were taken per test area. The Chromameter provided L*a*b* values, which were exported to Excel. L* is a measure of lightness, and a* and b* are measures of green‐red and blue‐yellow colour components, respectively.^26^, ^27^, ^28^ Staining levels were calculated using the Commission Internationale de L'éclairage L*a*b* method.^28^ ∆L*, ∆a*, ∆b* values were calculated in Excel (L*a*b* values after product exposure minus the untreated control L*a*b* values). ∆E (total difference) was calculated using the following equation:

$$\Delta E=\sqrt{({\left(\Delta L*\right)}^{2}+{\left(\Delta a*\right)}^{2}+{\left(\Delta b*\right)}^{2}}$$

### 2.7 Lipid sample collection

Five to 10 min after product exposure, a sample was collected for lipid analysis. The untreated control sample was collected prior to any product exposure. Swab sampling was performed on each test area by a trained technician, according to the instructions provided in the kit supplied by Synelvia. The kits contained two sterile cotton swabs and 500 μl of sampling buffer (Synelvia proprietary information; a mixture of surfactant and antioxidant/chelating agents) in an Eppendorf tube. Cotton swabs were wet by soaking in the sampling buffer and applied to the skin surface. Pressure was applied to the swab as it was moved over the whole skin test area for 45 s. The swab was cut with scissors and placed into the Eppendorf tube. This procedure was repeated with the second swab, which was then placed in the same Eppendorf tube. Samples were stored directly on wet ice and stored in a −20°C freezer within 2 h of sampling, before being shipped on dry ice to Synelvia SAS (Toulouse, France) for analysis.

### 2.8 SQ and SQ monohydroperoxide analysis by liquid chromatography‐mass spectrometry

For SQ quantification, lipids were extracted from swabs by liquid/liquid extraction.^29^ Briefly, samples were resuspended in 50 μl of dichloromethane (Honeywell) and an Agilent 6890N gas chromatography (GC) unit coupled to an Agilent 5975 Mass Selective Detector mass spectrometer^29^ used for analysis. The concentration of SQ in each sample was normalized to the total protein content and the surface area of exposed skin; values were expressed as μg/mg and μg/cm^2^, respectively. SQOOH was quantified using a double liquid/liquid extraction and UltiMate 3000 (Dionex) liquid chromatography system coupled to a MSQ Plus detector (Fisher Scientific).^29^ The concentration of SQOOH in the swabs was normalized to the total protein content and the surface area of exposed skin; values were expressed as ng/mg and ng/cm^2^, respectively. The ratio of SQ to SQOOH was determined by dividing the SQOOH values by the SQ value. To obtain convenient numbers, the ratio was calculated as ng SQOOH divided by μg SQ.

### 2.9 Malondialdehyde analysis by GC/MS

Samples were treated by acid hydrolysis and O‐(2,3,4,5,6‐pentafluoro‐benzyl) (PFB) hydroxylamine hydrochloride derivation performed, followed by liquid/liquid extraction.^29^ MS was performed as described by Curpen et al using a negative ion chemical ionization source.^29^ The concentration of MDA in each sample was normalized to the total protein content and to the surface area of exposed skin; values were expressed as ng/mg and ng/cm^2^, respectively.

### 2.10 Catalase analysis by fluorescence

The concentration of catalase in the sebum samples was calculated using a colorimetric method set up by Synelvia. The technique measures the level of hydrogen peroxide (H_2_0_2_) in samples, which is inversely proportional to the activity of catalase. The concentration of catalase in each sample was normalized to the total protein content and the surface area of exposed skin; values were expressed as UI/mg and UI/cm^2^, respectively.

### 2.11 Total protein estimation

Total protein content in the swab samples was assessed using two methods based on the Bicinchoninic Acid (BCA) assay and Coomassie blue reagent (Bradford) assay. The kit for BCA was supplied by Euromedex (Souffelweyersheim, Frankreich) and the method recommended by the manufacture used (Bio Basic Inc, Markham, Canada). Samples were assessed at 562 nm using a plate reader (Spark; Tecan Group Ltd., Männedorf, Switzerland). The Coomassie blue reagent kit was supplied by Abcam and the method recommended by the manufacture used. Samples were assessed at 595 nm using a plate reader (Spark).

### 2.12 Statistical data analysis

Descriptive statistics (*n*, mean, standard deviation) and 95 % confidence limits were calculated. A significance level of 0.05 (alpha) was chosen for statistical analysis. Due to the explorative character of the study, no adjustment for multiplicity was done. For all instrumental parameters, comparisons of treatments were performed on raw data with a paired *t* test. The computation of statistical data was carried out with commercially available statistics software (SAS Software.9.4 [2019], SAS Institute Inc., Cary, NC, USA).

## 3 RESULTS

Seven female (70%) and three male (30 %) subjects, aged 52.8 ± 9.8 years (mean ± standard deviation), completed the study. No adverse events were documented. In the case of skin discolouration, statistically significant differences were observed between CS, glo and ePen 3 exposure (Table 2). L* values (white to black) lowered following CS exposure, signifying a darkening of the skin. CS L* value was also significantly lower than glo (*p* < 0.001), ePen 3 (*p* = 0.003) and untreated control (*p* < 0.001) values. The L* values for glo, ePen 3 and untreated control were comparable. CS a* values (green to red) were significantly higher, signifying a reddening of the skin, when compared glo (*p* = 0.036) and ePen 3 (*p* = 0.022) values. CS and untreated control a* values were comparable; ePen 3, glo and untreated control a* values were also comparable. Following CS exposure, b* values (blue to green) were higher, signifying a yellowing of the skin. CS b* values were also significantly higher than glo, ePen 3 and untreated control values (all *p* < 0.001). Again ePen 3, glo and untreated control b* values were comparable.

**TABLE 2:** L*, a* and b* values following product exposure. Mean and standard deviation L*, a* and b* values following skin exposure to cigarette smoke, glo or ePen 3 aerosols and untreated control

				*p*‐values of comparison to control and products		
		Mean	SD	untreated	ePen 3	Glo
L* (white to black)	**Untreated**	69.12	3.66	–	–	–
	**ePen 3**	69.41	4.33	0.480	–	–
	**Glo**	69.30	3.56	0.730	0.823	–
	**Cigarette**	66.79	2.57	<0.001	0.003	<0.001
a* (green to red)	**Untreated**	7.43	1.57	–	–	–
	**ePen 3**	6.92	1.89	0.174	–	–
	**Glo**	7.32	1.88	0.863	0.430	–
	**Cigarette**	8.28	0.95	0.114	0.022	0.036
b* (blue to yellow)	**Untreated**	16.32	2.41	–	–	–
	**ePen 3**	15.79	2.92	0.187	–	–
	**Glo**	15.72	2.72	0.231	0.748	–
	**Cigarette**	20.72	1.91	<0.001	<0.001	<0.001

**FIGURE 2:**
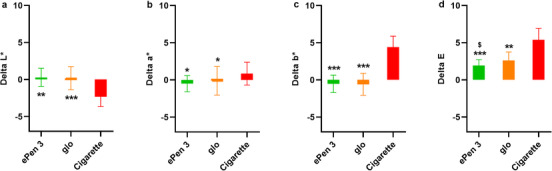
∆L*, ∆a*, ∆b* and ∆E values following product exposure. Mean and standard deviation ∆L*, ∆a*, ∆b* and ∆E values following exposure to cigarette smoke, glo or ePen 3 aerosols. *p ≤ 0.05, **p ≤ 0.01, ***p ≤ 0.001 for ePen 3 and glo aerosol values compared to cigarette smoke values. $ *p* ≤ 0.05 for ePen 3 aerosol values compared to glo aerosol values

∆L*, ∆a* and ∆b* values (Table 3, Figure 2) were calculated by subtracting untreated control L*, a* and b* values from CS, glo and ePen 3 post‐product exposure L*, a* and b* values (Table 2). ∆E values, the total colour difference from the untreated control, were calculated using the equation described above. CS ∆L*, ∆a* and ∆b* values were all significantly different than glo and ePen 3 values (see Table 3 for *p* values). The CS ∆E value was significantly higher than glo (*p* < 0.001) and ePen 3 (*p* = 0.002) values; the glo ∆E value was also significantly higher than the ePen 3 (*p* = 0.049) value.

**TABLE 3:** ∆L*, ∆a*, ∆b* and ∆E values following product exposure. Mean and SD ∆L*, ∆a*, ∆b* and ∆E values following exposure to cigarette smoke, glo or ePen 3 aerosols

				*p*‐values of comparison to	
		Mean	SD	ePen 3	Glo
∆L* **(white to black)**	**ePen 3**	0.29	1.23	–	–
	**Glo**	0.18	1.57	0.823^.^	–
	**Cigarette**	−2.33	1.32	0.003	<0.001
∆a* **(green to red to green)**	**ePen 3**	−0.51	1.09	–	–
	**Glo**	−0.11	1.93	0.43	–
	**Cigarette**	0.85	1.54	0.022	0.036
∆b* **(blue to yellow)**	**ePen 3**	−0.53	1.17	–	–
	**Glo**	−0.60	1.48	0.748	–
	**Cigarette**	4.40	1.49	<0.001	<0.001
**∆E** **(total colour difference from control)**	**ePen 3**	1.93	0.78	–	–
	**Glo**	2.61	1.14	0.049	–
	**Cigarette**	5.39	1.54	<0.001	0.002

**FIGURE 3:**
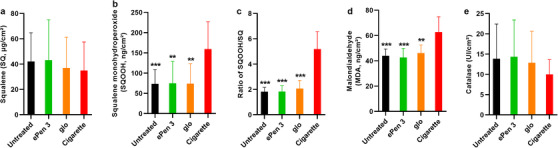
Skin biomarker levels following product exposure. SQ, SQOOH, SQOOH/SQ ratio, MDA and catalase mean and standard deviation values following exposure to cigarette smoke, glo or ePen 3 aerosols and for untreated controls. *p ≤ 0.05, **p ≤ 0.01, ***p ≤ 0.001 for ePen 3 and glo aerosol values compared to cigarette smoke values

Differences were observed in the levels of some of the skin biomarkers assessed (Table 4 and Figure 3). SQOOH levels increased following CS exposure, and levels were significantly higher than glo (*p* = 0.005), ePen 3 (*p* = 0.009) and untreated control (*p* = 0.001) levels, whereas SQ levels were comparable in all samples. The ratio of SQOOH/SQ (ng/μg) was significantly higher following CS exposure compared to glo, ePen 3 and control (all *p* < 0.001). CS also significantly increased MDA values compared to glo (*p* = 0.003), ePen 3 (*p* < 0.001) and untreated control (*p* = 0.001). Catalase values were comparable between all treatment groups.

**TABLE 4:** Skin biomarker levels following product exposure. SQ, SQOOH, SQOOH/SQ ratio, MDA and catalase mean and standard deviation values following exposure to cigarette smoke, glo or ePen 3 aerosols and for untreated controls

				*p*‐values of comparison to products and control		
		Mean	SD	Untreated	ePen 3	Glo
**Squalene (SQ) (μg/cm^2^)**	**Untreated**	42.15	22.64	–	–	–
	**ePen 3**	43.10	31.85	0.873	–	–
	**Glo**	36.97	24.29	0.268	0.093	–
	**Cigarette**	34.95	22.54	0.192	0.441	0.801
**Squalene monohydroperoxide (SQOOH)** **(ng/cm^2^)**	**Untreated**	73.35	35.21	–	–	–
	**ePen 3**	74.89	54.25	0.871	–	–
	**Glo**	73.80	49.34	0.957	0.835	–
	**Cigarette**	159.45	67.26	0.001	0.009	0.005
**Ratio of SQOOH/SQ** **(ng/μg)**	**Untreated**	1.83	0.34	–	–	–
	**ePen 3**	1.84	0.46	0.907	–	–
	**Glo**	2.07	0.65	0.224	0.054	–
	**Cigarette**	5.19	1.38	<0.001	<0.001	<0.001
**Malondialdehyde (MDA)** **(ng/cm^2^)**	**Untreated**	43.94	5.39	–	–	–
	**ePen 3**	42.69	7.16	0.572	–	–
	**Glo**	46.10	6.46	0.266	0.154	–
	**Cigarette**	62.80	12.02	= 0.001	<0.001	0.003
**Catalase** **(UI/cm^2^)**	**Untreated**	13.83	8.59	–	–	–
	**ePen 3**	14.36	9.06	0.617	–	–
	**Glo**	12.87	7.77	0.377	0.061	–
	**Cigarette**	10.01	3.63	0.067	0.067	0.152

## 4 DISCUSSION

ECs have been commercially available at a global scale for nearly 20 years. THPs were originally launched in Japan 5−6 years ago and are currently available in 54 countries.^30^ Scientific data from peer reviewed publications, including laboratory^4^, ^5^, ^6^, ^7^, ^8^, ^9^ and clinical assessments,^10^, ^11^, ^12^, ^13^, ^16^ have confirmed reduced responses in cells and reduced levels of biomarkers in consumers when THPs and ECs have been compared to CS. Following an independent review of these studies, a number of regulatory bodies have stated that THPs and ECs hold great potential for reducing the risk associated with cigarette smoking.^31^, ^32^, ^33^, ^34^, ^35^

In this study, we assessed the effect of CS, THP and EC aerosols by analysing skin colour and the activation of a number skin biomarkers. CS exposure resulted in a significant reddening and yellowing of skin. THP exposure resulted in some colour change compared to EC exposure. However, the values were significantly lower than CS, and in the blue and green colour space rather than the red and yellow space observed with CS. ECs did not result in a measurable colour change, and responses were comparable to untreated controls. The colour change induced by THPs could be due to the tobacco contained in the tobacco rod; heating could release particles that diffuse and sediment onto the skin. When wallpaper was exposed and then aged for 28 days,^26^ THP aerosols induced a higher level of staining than ECs. Glycerol could account for THPs and ECs ∆a* and ∆b* colour values being negative compared to the positive values after CS exposure, which suggesting that THP and EC aerosols could induce different responses in skin than CS. CS has been associated with the yellowing of fingernails and facial hair as well as greying of facial skin.^17^

Exposure of skin to CS can cause localised oxidative stress, resulting in the oxidation of lipids, proteins and DNA. Continued CS exposure can affect barrier integrity of the skin and cause connective tissue degeneration, leading to wrinkle formation.^17^, ^18^, ^19^, ^20^, ^21^ Twin studies, involving one smoker and one non‐smoker, have highlighted CS‐induced changes to the skin.^20^, ^36^ The degree of skin damage/aging is also thought to correlate with the number of cigarettes smoked per day and years of smoking.^37^ This study assessed the activation of sebum SQ, SQOOH, MDA and catalase biomarkers. These biomarkers function in skin homeostasis, are part of skin oxidative stress/damage pathways and are known to be modified by CS. SQ is a component of human sebum and is converted to SQOOH upon exposure to reactive oxygen.^38^ MDA is a subsequent metabolite and is formed following lipid peroxidation.^39^ A correlation between years of smoking and the levels of serum MDA has been suggested.^19^ In this study, the sebum lipid peroxidation product SQOOH increased after CS exposure, indicating a higher level of oxidation of skin surface sebum lipids and resulting in a higher SQOOH/SQ ratio. The barrier lipid peroxidation product MDA also increased following CS exposure, indicating a higher level of oxidative stress in the skin and oxidation of lipids of the stratum corneum. Although no significant differences were found, CS was observed to decrease the level of the anti‐oxidative enzyme catalase compared to the other treatments. SQ levels were comparable between all treatments. THP or EC exposure had no effect on the levels of the biomarkers assessed; levels were comparable to untreated controls. Variability was high for SQ and catalase levels, which could be due to low subject numbers. The colorimetric catalase method may also have caused variability. The analytical methods used in this study are aligned to a recent publication that assessed changes in the level of SQ, SQOOH and MDA following exposure of skin to ozone and dust.^29^

Differences in skin colour and biomarker levels following THP and EC exposure are probably due to the difference between CS and aerosols from THPs and ECs. As the tobacco in a cigarette burns, over 7000 chemicals, including a number of known toxicants, are produced.^40^ CS also contains reactive oxygen species and free radicals that can interact directly with the skin, resulting in oxidative stress and secondary oxidative events such as lipid peroxidation.^41^ In contrast, THPs and ECs produce chemically less complex aerosols with significantly reduced levels of toxicants and particles.^1^, ^2^, ^3^, ^4^, ^42^, ^43^ Similar to the reduced responses in subjects following the clinical assessment of THP and EC products,^10^, ^11^, ^12^, ^13^, ^16^ we observed significantly reduced responses in skin following THP and EC exposure, compared to CS.

The methods used in this study are an amalgamation of a laboratory method developed for assessing enamel sample staining^27^ ^,^ ^44^ and a clinical method developed to assess topical cosmetics, in which CS smoke is used as a surrogate for environmental pollution.^24^, ^25^ The main advantage of this clinical method is the small number of subjects required. Moreover, the developed method is not restricted to assessing tobacco and nicotine products; it could easily be adapted for the assessment of other aerosols or environmental pollutants and also used for cosmetic assessment. Standard methods of exposure, as used in this study, would enable data to be compared between laboratories and between cosmetic products.

The small volume of the experimental chamber enabled accelerated skin responses to be assessed. Specific puffing regimes were used for exposure,^45^, ^46^ which delivered 55 ml of each aerosol to a 3‐cm diameter isolated area of skin every 30 s. The total exposure time was 16 min, puff number was 32 and a total of 1760 ml of concentrated aerosol was delivered; a significantly higher concentration of aerosol than a consumer would be exposed to in a standard room per day. Data generated from this exposure chamber could potentially be extrapolated to a larger space/room to understand the long‐term impact of THP and EC aerosols on the skin.

A recent consumer study which aimed to understand Japanese consumers’ motivation for switching to a THP highlighted reduced harm to the consumer, hygiene and social considerations.^47^ Other studies have also highlighted potential hygiene benefits of THPs and ECs: reduced staining of tooth enamel^26^ and household materials.^26^ Compared to a cigarette, THP use also results in reduced hand, clothes and hair odour as well as reduced toxicants in a room.^48^ The data from the current study add to the weight of evidence that THPs and ECs have cosmetic and hygiene benefits for consumers compared to smoking: the data suggest that EC and THP aerosols have less impact on consumers’ skin than CS. If THPs or ECs are used indoors, there could also be a benefit to bystanders’ skin compared to CS.

A limitation of this study is that the experimental method delivered mainstream CS, but neither side‐stream smoke that is emitted from a smouldering cigarette between puffs nor exhaled smoke. THPs and ECs do not burn tobacco or produce side stream aerosols because aerosols are only released after puffing on the THP consumable/stick or EC device mouthpiece by the consumer. Our method may therefore result in overrepresentation of THP and EC responses and under‐representation of CS responses. Significant differences were nevertheless observed between the products being assessed.

## 5 CONCLUSIONS

CS exposure results in a higher level of skin discolouration, oxidation of sebum lipids and oxidation of skin barrier lipids. In contrast, THP and CS exposure results in responses comparable to untreated controls. The data generated in this pilot clinical assessment suggest that THPs and ECs may have both hygiene and cosmetic benefits for consumers who switch from cigarettes to exclusive use of THPs or ECs. Further studies are required to assess the long‐term impact of consumers’ skin following the exclusive use of a THP or EC.

## CONFLICT OF INTEREST

This study was funded by British American Tobacco (BAT) R&D, Southampton. Experimental work was performed at proDERM GmbH and Synelvia SAS. All authors are employees of BAT, proDERM or Synelvia.

## Supporting information

Supporting information

## References

[1] Margham J , McAdam K , Forster M , Liu C , Wright C , Mariner D , et al. Chemical composition of aerosol from an e‐cigarette: a quantitative comparison with cigarette smoke. Chem Res Toxicol. 2016;29(10):1662–78. 27641760 10.1021/acs.chemrestox.6b00188

[2] Forster M , Fiebelkorn S , Yurteri C , Mariner D , Liu C , Wright C , et al. Assessment of novel tobacco heating product THP10 Part 3: comprehensive chemical characterisation of harmful and potentially harmful aerosol emissions. Regul Toxicol Pharmacol. 2018;93:14–33 29080848 10.1016/j.yrtph.2017.10.006

[3] Cunningham A , McAdam K , Thissen J , Digard H . The evolving e‐cigarette: comparative chemical analyses of e‐cigarette vapor and cigarette smoke. Front Toxicol. 2020;2:7. 10.3389/ftox.2020.586674 PMC8915913 35296117

[4] Schaller JP , Keller D , Poget L , Pratte P , Kaelin E , Mchugh D , et al. Evaluation of the tobacco heating system 2.2. part 2: chemical composition, genotoxicity, cytotoxicity, and physical properties of the aerosol. Regul Toxicol Pharmacol. 2016;81(2):S27–47. 27720919 10.1016/j.yrtph.2016.10.001

[5] Thorne D , Crooks I , Hollings M , Seymour A , Meredith C , Gaca M . The mutagenic assessment of an electronic‐cigarette and reference cigarette smoke using the Ames assay in strains TA98 and TA100. Mutat Res. 2016;812:29–38. 10.1016/j.mrgentox.2016.10.005 27908385

[6] Zanetti F , Titz B , Sewer A , Lo Sasso G , Scotti E , Schlage WK , et al. Comparative systems toxicology analysis of cigarette smoke and aerosol from a candidate modified risk tobacco product in organotypic human gingival epithelial cultures: a 3‐day repeated exposure study. Food Chem Toxicol. 2017;101:15–35. 28025120 10.1016/j.fct.2016.12.027

[7] Thorne D , Hollings M , Seymour A , Adamson J , Dalrymple A , Ballantyne M , et al. Extreme testing of undiluted e‐cigarette aerosol in vitro using an Ames air‐agar‐interface technique. Mutat Res Genet Toxicol Environ Mutagen. 2018;828:46–54. 29555064 10.1016/j.mrgentox.2018.01.008

[8] Thorne D , Breheny D , Proctor C , Gaca M . Assessment of novel tobacco heating product THP1.0. Part 7: comparative in vitro toxicological evaluation. Regul Toxicol Pharmacol. 2018;93:71–83. 29080850 10.1016/j.yrtph.2017.08.017

[9] Thorne D , Whitwell J , Clements J , Walker P , Breheny D , Gaca M . The genotoxicological assessment of a tobacco heating product relative to cigarette smoke using the *in vitro* micronucleus assay. Toxicol Rep. 2020;7:1010–9 32874925 10.1016/j.toxrep.2020.08.013 PMC7451629

[10] Cravo AS , Bush J , Sharma G , Savioz R , Martin C , Craige S , et al. A randomised, parallel group study to evaluate the safety profile of an electronic vapour product over 12 weeks. Regul Toxicol Pharmacol. 2016;81(1):S1–14. 27769828 10.1016/j.yrtph.2016.10.003

[11] Martin F , Talikka M , Ivanov NV , Haziza C , Hoeng J , Peitsch MC . Evaluation of the tobacco heating system 2.2. Part 9: application of systems pharmacology to identify exposure response markers in peripheral blood of smokers switching to THS2.2. Regul Toxicol Pharmacol. 2016;81(2):S151–7. 27845159 10.1016/j.yrtph.2016.11.011

[12] Shahab L , Goniewicz ML , Blount BC , Brown J , Mcneill A . Nicotine, carcinogen, and toxin exposure in long‐term e‐cigarette and nicotine replacement therapy users: a cross‐sectional study. Ann Intern Med. 2017;166(6):390–400. 28166548 10.7326/M16-1107 PMC5362067

[13] Gale N , McEwan M , Eldridge AC , Fearon IM , Sherwood N , Bowen E , et al. Changes in biomarkers of exposure on switching from a conventional cigarette to tobacco heating products: a randomized, controlled study in healthy Japanese subjects. Nicotine Tob Res. 2019;21(9):1220–7. 29912406 10.1093/ntr/nty104 PMC6698948

[14] Makena P , Liu G , Chen P , Yates CR , Prasad GL . Urinary leukotriene E4 and 2,3‐dinor thromboxane B2 are biomarkers of potential harm in short‐term tobacco switching studies. Cancer Epidemiol Biomarkers Prev. 2019;28(12):2095–105. 31558507 10.1158/1055-9965.EPI-19-0342

[15] Round EK , Chen P , Taylor AK , Schmidt E . Biomarkers of tobacco exposure decrease after smokers switch to an e‐cigarette or nicotine gum. Nicotine Tob Res. 2019;21(9):1239–47. 30202883 10.1093/ntr/nty140 PMC6698949

[16] Gale N , McEwan M , Camacho OM , Hardie G , Murphy J , Proctor CJ . Changes in biomarkers of exposure on switching from a conventional cigarette to the glo tobacco heating product: A randomized, controlled ambulatory study. Nicotine Tob Res. 2020; 23:584–91. 10.1093/ntr/ntaa135 PMC7885769 32776101

[17] Ortiz A , Grando SA . Smoking and the skin. Int J Dermatol. 2012;51:250–62. 22348557 10.1111/j.1365-4632.2011.05205.x

[18] Lykkesfeldt J . Malondialdehyde as biomarker of oxidative damage to lipids caused by smoking. Clin Chim Acta. 2007;380(1–2):50–8. 17336279 10.1016/j.cca.2007.01.028

[19] Attwa E , Swelam E . Relationship between smoking‐induced oxidative stress and the clinical severity of psoriasis. J Eur Acad Dermatol Venereol. 2011;25(7):782–7. 21039915 10.1111/j.1468-3083.2010.03860.x

[20] Prieux R , Eeman M , Rothen‐Rutishauser B , Valacchi G . Mimicking cigarette smoke exposure to assess cutaneous toxicity. Toxicol In Vitro. 2020;62:104664. 31669394 10.1016/j.tiv.2019.104664

[21] Soeur J , Belaïdi JP , Chollet C , Denat L , Dimitrov A , Jones C , et al. Photo‐pollution stress in skin: traces of pollutants (PAH and particulate matter) impair redox homeostasis in keratinocytes exposed to UVA1. J Dermatol Sci. 2017;86:162–9. 28153538 10.1016/j.jdermsci.2017.01.007

[22] Urbańska M , Nowak G , Florek E . Wpływ palenia tytoniu na starzenie sie skóry [cigarette smoking and its influence on skin aging]. Przegl Lek. 2012;69(10):1111–4. 23421102

[23] Ebajemito JK , McEwan M , Gale N , Camacho OM , Hardie G , Proctor CJ . A randomised controlled single‐centre open‐label pharmacokinetic study to examine various approaches of nicotine delivery using electronic cigarettes. Sci Rep. 2020;10:19980. 33235307 10.1038/s41598-020-76610-4 PMC7686355

[24] Bielfeldt S , Böhling A , Laing S , Hoppe C , Wilhelm KP . Environmental skin protection strategies – a new clinical testing method employing a cigarette smoke pollutant model. SOFW J. 2016;142(11);10–7.

[25] Bielfeldt S , Springmann G , Seise M , Wilhelm K‐P , Callaghan T . An updated review of clinical methods in the assessment of ageing skin ‐ new perspectives and evaluation for claims support. Int J Cosmet Sci. 2018;40(4):348–55. 30047989 10.1111/ics.12484

[26] Dalrymple A , Badrock TC , Terry A , Bean E‐J , Barber M , Hall PJ , et al. Development of a novel method to measure material surface staining by cigarette, e‐cigarette or tobacco heating product aerosols. Heliyon. 2020;6(9):e05012. 32995648 10.1016/j.heliyon.2020.e05012 PMC7511806

[27] Dalrymple A , Badrock TC , Terry A , Barber M , Hall PJ , Thorne D , et al. Assessment of enamel discoloration in vitro following exposure to cigarette smoke and emissions from novel vapor and tobacco heating products. Am J Dent. 2018;31(5):227–33. 30346667

[28] MdeS W , Takahashi MK , Kirsten GA , De Souza EM . Effect of cigarette smoke and whiskey on the color stability of dental composites. Am J Dent. 2010;23:4–8. 20437719

[29] Curpen S , Francois‐Newton V , Moga A , Hosenally M , Petkar G , Soobramaney V , et al. A novel method for evaluating the effect of pollution on the human skin under controlled conditions. Skin Res Technol. 2020;26(1):50–60. 31373064 10.1111/srt.12763

[30] Shapiro H . Burning issues: the global state of tobacco harm reduction. 2020. Available from https://gsthr.org/resources/item/burning‐issues‐global‐state‐tobacco‐harm‐reduction‐2020. Accessed 6 Dec 2020.

[31] Committees on toxicity, Carcinogenicity and Mutagenicity of Chemicals in Food, Consumer Products and the Environment (COT, COC and COM) . Statement on the toxicological evaluation of novel heat‐not‐burn tobacco products. 2017. Available from https://cot.food.gov.uk/sites/default/files/heat_not_burn_tobacco_statement.pdf. Accessed 19 Oct 2020.

[32] cot . Committee on toxicity of chemicals in food, consumer products and the environment (COT). Statement on the potential toxicological risks from electronic nicotine (and non‐nicotine) delivery systems (E(N)NDS – e‐cigarettes). 2020. Available from https://cot.food.gov.uk/sites/default/files/2020‐09/COT%20E%28N%29NDS%20statement%202020‐04.pdf. Accessed 17 Sep 2020.

[33] Food and Drug Administration . FDA permits sale of IQOS tobacco heating system through premarket tobacco product application pathway. 2019. Available from https://www.fda.gov/news‐events/press‐announcements/fda‐permits‐sale‐iqos‐tobacco‐heating‐system‐through‐premarket‐tobacco‐product‐application‐pathway. Accessed 19 Oct 2020.

[34] McNeill A , Brose LS , Calder R , Hitchman SC , Hajek P , McRobbie H . E‐cigarettes: an evidence update. A report commissioned by Public Health England. 2015. Available from https://www.gov.uk/government/uploads/system/uploads/attachment_data/file/457102/Ecigarettes_an_evidence_update_A_report_commissioned_by_Public_Health_England_FINAL.pdf. Accessed 19 Oct 2020.

[35] McNeill A , Brose LS , Calder R , Bauld L , Robson D . Evidence review of e‐cigarettes and heated tobacco products. A report commissioned by Public Health England. 2018. Available from https://assets.publishing.service.gov.uk/government/uploads/system/uploads/attachment_data/file/684963/Evidence_review_of_e‐cigarettes_and_heated_tobacco_products_2018.pdf. Accessed 19 Oct 2020.

[36] Doshi DN , Hanneman KK , Cooper KD . Smoking and skin aging in identical twins. Arch Dermatol. 2007;143(12):1543–6. 18087005 10.1001/archderm.143.12.1543

[37] Ortiz A , Grando SA . Smoking and the skin. Int J Dermatol. 2012;51(3):250–62. 22348557 10.1111/j.1365-4632.2011.05205.x

[38] Pham DM , Boussouira B , Moyal D , Nguyen QL . Oxidization of squalene, a human skin lipid: a new and reliable marker of environmental pollution studies. Int J Cosmet Sci. 2015;37(4):357–65. 25656265 10.1111/ics.12208

[39] Fitzmaurice PS , Tong J , Yazdanpanah M , Liu PP , Kalasinsky KS , Kish SJ . Levels of 4‐hydroxynonenal and malondialdehyde are increased in brain of human chronic users of methamphetamine. J Pharmacol Exp Ther. 2006;319(2):703–9. 16857724 10.1124/jpet.106.109173

[40] Perfetti TA , Rodgman A . The chemical components of tobacco and tobacco smoke. 2nd ed. Boca Raton, FL: CRC Press; 2013.

[41] Pandit VI , Phadke KM . Gaseous composition of cigarette smoke: effect on human health and air pollution. Indian J Public Health. 1973;17(1):16–8. 4786733

[42] Mallock N , Böss L , Burk R , Danziger M , Welsch T , Hahn H , et al. Levels of selected analytes in the emissions of “heat not burn” tobacco products that are relevant to assess human health risks. Arch Toxicol. 2018;92(6):2145–9. 29730817 10.1007/s00204-018-2215-y PMC6002459

[43] Tayyarah R , Long GA . Comparison of select analytes in aerosol from e‐cigarettes with smoke from conventional cigarettes and with ambient air. Regul Toxicol Pharmacol. 2014;70(3):704–10. 25444997 10.1016/j.yrtph.2014.10.010

[44] Dalrymple A , Bean EJ , Badrock TC , Weidman RA , Thissen J , Coburn S , et al. Enamel staining with e‐cigarettes, tobacco heating products and modern oral nicotine products compared with cigarettes and snus: an in vitro study. Am J Dent. 2021;34(1):3–9. 33544982

[45] Health Canada . 1999 Health Canada: determination of “tar”, nicotine and carbon monoxide in mainstream tobacco smoke. https://healthycanadians.gc.ca/en/open‐information/tobacco/t100/nicotine. Accessed 19 Oct 2020.

[46] CORESTA . CORESTA recommended method no 81. Routine analytical machine for e‐cigarette aerosol generation and collection – definitions and standard conditions. https://www.coresta.org/sites/default/files/technical_documents/main/CRM_81.pdf. Accessed 19 Oct 2020.

[47] Adamson J , Kanitscheider C , Prasad K , Camacho OM , Beyerlein E , Bhagavan YK , et al. Results from a 2018 cross‐sectional survey in Tokyo, Osaka and Sendai to assess tobacco and nicotine product usage after the introduction of heated tobacco products (HTPs) in Japan. Harm Reduct J. 2020;17(1):32. 32450856 10.1186/s12954-020-00374-3 PMC7249648

[48] Forster M , McAughey J , Prasad K , Mavropoulou E , Proctor C . Assessment of tobacco heating product THP1.0. Part 4: Characterisation of indoor air quality and odour. Regul Toxicol Pharmacol. 2018;93:34–51. 28989082 10.1016/j.yrtph.2017.09.017

