# Supplementary material for: A novel clinical method to measure skin staining reveals activation of skin damage pathways by cigarette smoke
Source: Skin Res Technol. 2021 Nov 10;28(1):162–70. doi: 10.1111/srt.13108 (PMC9299119; doi:10.1111/srt.13108)
Supplement: Supplementary file 1 — Supporting information [file SRT-28-162-s001.docx]

**Supplementary Table1: Inclusion and exclusion criteria for subjects**

| **Inclusion criteria** |
| --- |
| Female or male 21 to 60 years of age |
| Written Informed Consent to participate in the study |
| Willingness to actively participate in the study and to come to the scheduled visits |
| Understand and comply with the requirements of the study |
| Good general health as judged by the Investigator as based on their medical history |
| Healthy skin in the test area |
| No diseases or conditions which could affect the study or have an adverse impact on the subject including dermatitis, eczema, psoriasis, systemic inflammatory diseases |
| Have never smoked or have quit smoking within 3 months before the start of the study |
| **Exclusion criteria** |
| Female subjects: Pregnancy or lactation |
| Drug or alcohol addiction |
| AIDS, HIV or infectious hepatitis if known to the subjects |
| Conditions which exclude a participation or might influence the test reaction/evaluation |
| Participation in similar cosmetic and/or pharmaceutical studies |
| Cancer within the last 2 years if treated with chemotherapy and/or irradiation |
| Insulin-dependent diabetes mellitus |
| Medical history of dysplastic nevi or melanoma |
| Moles, tattoos, scars, irritated skin, hairs, etc. at the test area that could influence the investigation |
| Any topical medication at the test area within 3 days of the start of the study |
| Systemic therapy with immuno-suppressive drugs (e.g. corticosteroids) and/or antihistamines (e.g. antiallergics) within 7 days prior to the start of the study |
| Systemic therapy with analgetics (e.g. diclophenac, except for minor pain relief medicine, like acetylsalicylic acid or paracetamol) within 3 days prior to the start of the study |
| Intake of dietary supplements with anti-oxidative effects (e.g. vitamin C, beta-carotene, vitamin E, selen) within 3 months prior to the start of the study |
| History of kidney disease |
| Current use or use of any antibiotics within 14 days prior to the start of the study |
| Current use or use of anti-inflammatory medication within 4 weeks prior to the start of the study. |
| Employees and immediate relatives of the tobacco industry or the clinical site |
